# A fixed intraoral nail biting habit-breaker appliance: A case report of a novel approach to prevent onychophagia

**DOI:** 10.15171/joddd.2019.027

**Published:** 2019-10-07

**Authors:** Shankhanil Dev, Ananya Pal, Shabnam Zahir, Gautam Kumar Kundu

**Affiliations:** ^1^Department of Pedodontics & Preventive Dentistry, Guru Nanak Institute of Dental Science and Research, Kolkata, West Bengal, India; ^2^Guru Nanak Institute of Dental Science and Research, Kolkata, West Bengal, India

**Keywords:** Appliance, habit-breaker, onychophagia

## Abstract

Onychophagia or nail biting is the performance of repetitive actions of biting one’s nails often to the level of mutilation of the nail beds. It is a compulsive act most often seen in adolescents but may continue into adulthood, leading to deleterious consequences. Often spurred by anxiety and stress, this oral habit is not so readily addressed by patients and in turn not very much treated by dentists or physicians. This case report describes successful treatment of an adolescent patient with a nail biting habit, with an innovative intraoral fixed habit-breaker appliance.

## Introduction

Nail biting or onychophagia is defined as the crossing of any digit from an individual’s lips.^1^ It is a “body-focused repetitive behavioral act,” i.e. it is habitual and difficult to suppress.^2^ It usually does not manifest before the age of 3 or 4 years.^2^ Prevalence varies from 20 to 33% in childhood.^2^ The frequency of this habit decreases by 18 years of age but may continue into adulthood.^2^ Males and females up to 10 years of age may equally perform the habit; thereafter it is seen more frequently in males.^3^ Onychophagia falls under impulse control disorder in the DSM-IV-R and is classified as an obsessive‒compulsive and related disorder in the DSM-5.^3^ Subjects with onychophagia can chew off nail beds, resulting in chronic scarring accompanied by red inflamed fingers.^3^ Nail biting is associated with some complications, including infection of the nail beds, notably onychomycosis and onycholysis.^6^ Deleterious dental effects are alveolar bone destruction, apical root resorption, chipping off incisal edges of anterior teeth, minor crowding, rotation, proclination of maxillary anterior teeth and temporomandibular joint dysfunction.^6^ Occasionally such habit might be so severe as to cause entrapment of bitten-off finger nails in the gingiva.^5^

Treatment modalities range from restriction of performance of this act through application of bitter tasting solutions on finger nails to psychotherapy and pharmacotherapy.^6^

A case is presented here, where nail biting habit in a pediatric patient was successfully treated by an intraoral fixed deterrent appliance.

## Case Report

**Figure 1:**
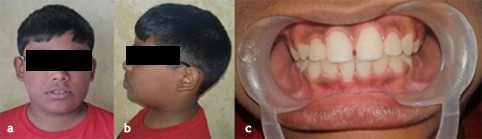
Preoperative: a) Extraoral frontal view; b) Extraoral lateral view; c) Intraoral view.

**Figure 2:**
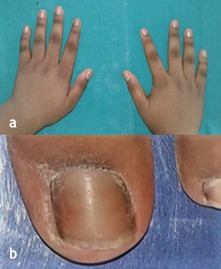
Preoperative view of a) fingernails; b) mutilated toe nail.

An 11-year-old boy reported to the Department of Pedodontics and Preventive Dentistry, with his parents complaining of their boy being engaged in biting of nails of fingers and toes since 9 years of age. General examination of the boy revealed mutilated finger nails (Figure 2a) and toe nails (Figure 2b). Extraoral examination revealed a dolichocephalic face with short upper lip (Figure 1a). Intraoral examination showed bimaxillary protrusion with Angle’s class I malocclusion bilaterally (Figure 1c). Radiographic examination revealed protruded maxillary and mandibular anteriors (Figure 4a and 4b). Counseling was attempted initially but the patient was not sufficiently motivated. After obtaining informed consent, it was decided to insert an interceptive fixed intraoral deterrent appliance. Efficacy of this treatment was decided to be ascertained by comparison of scores obtained in a questionnaire (Table 1) answered pre- and post-operatively. This questionnaire was based on the Massachusetts General Hospital hair-pulling scale.^8^ It consisted of seven questions having options with grades from zero to four. The minimum summed up score was zero and the maximum was 28. The patient was asked to fill up the questionnaire. Before fixation of the appliance, the grades summed up to 11.

**Table 1:** The nail-biting questionnaire

**Question 1**	**Question 2**	**Question 3**	**Question 4**	**Question 5**	**Question 6**	**Question 7**
On an average day how often did you feel the urge to bite your nails?	On an average day how intense or strong were the urges to bite your nails?	On an average day how much control do you have over the urges to bite your nails?	On an average day how often did you actually bite your nails?	On an average day how often did you make an attempt to stop yourself from biting your nails?	On an average day how often were you successful at actually stopping yourself from biting your nails?	During the past week how uncomfortable did your nail biting make you feel?
0-No urge	0-No urge	0-Always in control	0-Never	0-No urges	0-Did not bite	0-Did not feel uncomfortable
1-Occassional urge	1-Mild	1-Most of the time	1-Occassionally	1-Almost all of the time	1-Almost all of the time	1-Vaguely uncomfortable
2-Often	2-Moderate	2-Some of the time	2-Often	2-Some of the time	2-Most of the time	2-Noticeably uncomfortable
3-Very often	3-Severe	3-Rarely	3-Very often	3-Rarely	3-Some of the time	3-Significantly uncomfortable
4-Near constant urges	4-Extreme	4-Never in control	4-Always	4-Never tried to resist the urges	4-Rarely able to resist	4-Intensely uncomfortable

**Figure 3:**
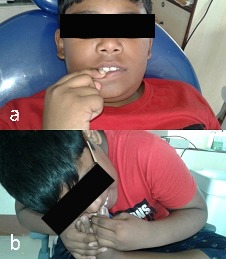
Patient in a) fingernail biting posture; b) toenail biting posture.

**Figure 4:**
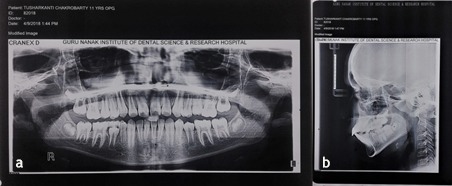
Preoperative: a) Panoramic view; b) Lateral cephalogram

**Figure 5:**
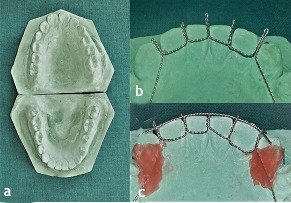
a) Preoperative maxillary and mandibular casts; b) The horizontal component of twisted ligature wires along with vertical extensions being fabricated on the lower cast; c) Additional horizontal component of twisted ligature wires placed along the incisal edges of the lower anterior teeth.

Maxillary and mandibular impressions were made in alginate to prepare working casts (Figure 5a). The appliance was fabricated with a modification of the design advocated by Marouane et al.^6^ It consisted of 26-gauge stainless steel round wires twisted around each other. These were adapted along the lingual surfaces of the mandibular anteriors in the cast. Vertical extensions arose from the wires lying interdentally (Figure 5b). Modification in this appliance framework was introduced by us, by adding a third horizontal assembly of twisted stainless-steel round wires resting on the incisal edges (Figure 5c).

**Figure 6:**
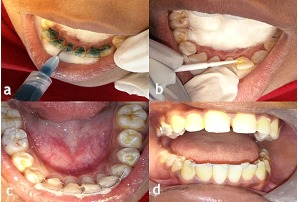
a) Etching the lingual surfaces of the lower anterior teeth; b) Application of a bonding agent on the etched teeth; c) Application of light-cured composite resin on the lower anterior teeth after fixing the appliance on the selected teeth; d) Intraoral frontal view of the appliance after being fixed on the selected teeth.

Oral prophylaxis followed by proper isolation was done intraorally. Following etching (Figure 6a) and application of a bonding agent (Figure 6b), the appliance was fixed in its desired position with light-cured composite resin (Figure 6c and 6d).

**Figure 7:**
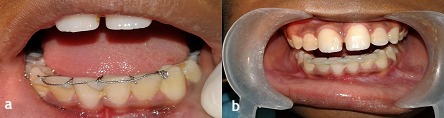
a) Intraoral frontal view after 3 weeks, showing shifting of the horizontal component labially, which was previously present along the incisal edges; b) The shifted horizontal component was fixed with light-cured composite resin in its new position.

The patient was recalled after 1 month. It was observed during that time that the horizontal component lying on the incisal edges had shifted labially (Figure 7a). The shifted horizontal component was fixed in its new position with light-cured composite resin (Figure 7b).

**Figure 8:**
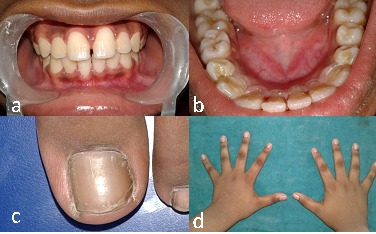
a) Intraoral frontal view after 2 months; b) Intraoral lingual view after 2 months; c) Appearance of neat toe nail after 2 months; d) Appearance of fingernails of both hands after 2 months, showing clean and neat nails.

The patient was recalled every 3 months for follow-up. Complete resolution and withdrawal of the habit was seen and finger and toe nails had clean and neat appearance at 6-month follow-up. The appliance was removed. The patient was again requested to answer the same questionnaire. The summed-up grades turned out to be 2 compared to the previous score of 11, indicating success in the treatment with this intraoral appliance. The patient is still under follow-up for a further period of 6 months.

## Discussion

In 1950, Massler and Malone theorized that persons under tension bite their nails.^4^ Correlation of nail biting with regression to oral gratification on events of fatigue was postulated by Pierre.^4^ Various modalities are available to treat onychophagia. Among them the psychological and dermatological aspects remain the prime focus.^6^ The appliance described here serves the purpose of providing a mechanical restraint as well as a reminder to the patient about this deleterious habit. Aversive stimulus is quite often effective in managing nail biting habit.^6^ It is usually achieved through painting bitter solutions on nails.^6^ By punishing every attempt of nail biting, this fixed appliance works as an aversion-based behavioral modification technique.^6^ Reinforcement learning forms a core element of aversion technique but it also constitutes a reminder that is self-terminating, requiring reactivation.^6^

As suggested by Koritzky and Yechiam, the application of constantly present reminders widens the target population benefitting from reminders during the period of behavior modification.^6^ Here, the fixed appliance serves as a mechanical hindrance in addition to serving as a reminder of one’s aim to avoid biting nails.

Nail biting is performed in four phases.^6^ Firstly the finger is inspected visually and palpated by the other finger followed by placement in front of the mouth. Then the mandible is placed in a laterotrusive edge-to-edge contact position. Thirdly, the fingers are quickly tapped against the front teeth, accompanied by a series of quick spasmodic biting actions. Subsequently, fingernails are pressed tightly against the incisal edges, followed by withdrawal of the fingers.

This intraoral appliance prevents the third phase, i.e. the biting phase. The horizontal component, placed along the incisal edges restricts the placement of the nails between the incisal surfaces of the maxillary and mandibular anterior teeth. At two-month follow-up, there was complete resolution of the habit along with the presence of neat and normal appearance of finger and toe nails. No complaint was obtained from the patient regarding difficulty in eating or speech due to the appliance. Factors such as frequency, duration, intensity of the habit and cooperation of the patient are essential for the treatment to be successful.^6^

## Conclusion

Onychophagia is a habit that exists in today’s stress- burdened society. Timely and proper treatment can intercept this habit, which will prove beneficial in the long run. An innovative fixed intraoral appliance like the one, described in this case report would effectively train the individual to quit the deleterious habit and help in self-reflection of one’s actions in order to live a healthy life.

## Authors’ Contributions

SD contributed towards carrying out the treatment on the patient as well as in writing of the manuscript. AP contributed towards capturing clinical images of the treatment. SZ contributed towards conceiving the idea of the treatment planning as well as in writing of the manuscript. GKK contributed towards supervising the treatment procedure as well as in capturing images.

## Acknowledgments

The authors would like to thank all the staff and postgraduate students of the Departments of Pedodontics and Preventive Dentistry, Guru Nanak Institute of Dental Science and Research, Kolkata, West Bengal, India.

## Funding

Not applicable

## Competing Interests

The authors declare no competing interests with regards to the authorship and/or publication of this article

## Ethics Approval

Not applicable. Consent was obtained to publish pictures of the individual involved in the case report of this article. Identity of the individual has been masked in the pictures.
